# Differential Expression of Genes Related to Innate Immune Responses in Ex Vivo Spinal Cord and Cerebellar Slice Cultures Infected with West Nile Virus

**DOI:** 10.3390/brainsci9010001

**Published:** 2018-12-24

**Authors:** Parminder J. S. Vig, Deyin Lu, Amber M. Paul, Ram Kuwar, Maria Lopez, Dobrivoje S. Stokic, A. Arturo Leis, Michael R. Garrett, Fengwei Bai

**Affiliations:** 1 Departments of Neurology, University of Mississippi Medical Center, Jackson, MS 39216, USA; dlu@umc.edu (D.L.); mlopez@umc.edu (M.L.); fengwei.bai@usm.edu (F.B.); 2 Biochemistry, University of Mississippi Medical Center, Jackson, MS 39216, USA; 3 Neurobiology & Anatomical Sciences, University of Mississippi Medical Center, Jackson, MS 39216, USA; dstokic@mmrcrehab.org; 4 Department of Biological Sciences, University of Southern Mississippi, Hattiesburg, MS 39406, USA; ambermariepaul@gmail.com; 5 Virginia Commonwealth University, Richmond, VA 23284, USA; rbkuwar@gmail.com; 6 Methodist Rehabilitation Center, Jackson, MS 39216, USA; aleis@mmrcrehab.org; 7 Experimental Therapeutics and Pharmacology, University of Mississippi Medical Center, Jackson, MS 39216, USA; mrgarrett@umc.edu

**Keywords:** West Nile, interferons, cerebellum, spinal cord, cultures, gene expression, astrogliosis

## Abstract

West Nile virus (WNV) infection results in a spectrum of neurological symptoms, ranging from a benign fever to severe WNV neuroinvasive disease with high mortality. Many who recover from WNV neuroinvasive infection present with long-term deficits, including weakness, fatigue, and cognitive problems. While neurons are a main target of WNV, other cell types, especially astrocytes, play an important role in promoting WNV-mediated central nervous system (CNS) damage. Conversely, it has been shown that cultured primary astrocytes secrete high levels of interferons (IFNs) immediately after WNV exposure to protect neighboring astrocytes, as well as neurons. However, how intrinsic responses to WNV in specific cell types and different regions of the brain modify immune protection is not fully understood. Here, we used a mouse ex vivo spinal cord slice culture (SCSC) and cerebellar slice culture (CSC) models to determine the innate immune responses specific to the CNS during WNV infection. Slices were prepared from the spinal cord and cerebellar tissue of 7–9-day-old mouse pups. Four-day-old SCSC or CSC were infected with 1 × 10^3^ or 1 × 10^5^ PFU of WNV, respectively. After 12 h exposure to WNV and 3 days post-infection in normal growth media, the pooled slice cultures were processed for total RNA extraction and for gene expression patterns using mouse Affymetrix arrays. The expression patterns of a number of genes were significantly altered between the mock- and WNV-treated groups, both in the CSCs and SCSCs. However, distinct differences were observed when CSC data were compared with SCSC. CSCs showed robust induction of interferons (IFNs), IFN-stimulated genes (ISGs), and regulatory factors. Some of the antiviral genes related to IFN were upregulated more than 25-fold in CSCs as compared to mock or SCSC. Though SCSCs had twice the number of dysregulated genes, as compared CSCs, they exhibited a much subdued IFN response. In addition, SCSCs showed astrogliosis and upregulation of astrocytic marker genes. In sum, our results suggest that early anti-inflammatory response to WNV infection in CSCs may be due to large population of distinct astrocytic cell types, and lack of those specialized astrocytes in SCSC may make spinal cord cells more susceptible to WNV damage. Further, the understanding of early intrinsic immune response events in WNV-infected ex vivo culture models could help develop potential therapies against WNV.

## 1. Introduction

West Nile virus (WNV) is a single-stranded RNA flavivirus that has a natural life cycle between birds and mosquitoes [1,2]. The spectrum of WNV infection in humans can range from a flu-like febrile illness to acute flaccid paralysis, meningitis, or encephalitis [3,4]. Neuroinvasive WNV infection has a high mortality rate, and many who recover present with long-term symptoms for years [5]. The most debilitating is an acute asymmetric poliomyelitis-like weakness with or without signs of viremia or meningoencephalitis [2,6]. Recent reports suggest a link between West Nile infection and myasthenia gravis, as observed in patients with no prior history of myasthenia gravis that developed this autoimmune disorder 3 to 7 months after recovery from neuroinvasive WNV disease [7,8]. A post-infectious pro-inflammatory state was hypothesized to contribute to the long-term lingering symptoms and to promote autoimmune disorders.

Histopathologic examination of brain and spinal cord tissues from nine victims of WNV neuroinvasive disease showed perivascular inflammation, microglial nodules, neuronophagia, and variable necrosis and neuronal loss [9]. These changes were dominant in the deep nuclei of the brain and anterior horns of the spinal cord. Focal demyelination, gliosis, and occasional perivascular infiltrates were also seen among patients with prolonged clinical courses [10]. Leis et al. [11] examined the serum and CSF of WNV patients who first had symptoms 2–18 days prior to sampling, and found elevated levels of glial fibrillary acidic proteins (GFAP)-SM 126 and S100B, suggesting astroglial activation [11,12].

The precise pathogenesis of WNV neuroinvasive disease is not yet clear [13]. Some of the in vitro studies showed capsid protein-induced apoptosis in the neurons through the activation of mitochondrial/caspase-9 and caspase-3 pathway during WNV infection [14], whereas other studies have shown that the infected neurons die due to the involvement and activation of immune cells such as cytotoxic T cells, as these neurons express higher amounts of MHC class I molecules [15,16]. However, a slice culture study of spinal cord showed that the CNS resident cells and microglia initiate a strong immune response against WNV infection by secreting large number of chemokines and cytokines [17].

One of the earliest innate host responses to a viral infection is the induction of type 1 interferon (IFN), especially IFN-α/β. This response is initiated by different types of cellular pathogen-recognizing receptors (PRRs), which can sense viral invasion [18,19]. They include several of the toll-like receptors (TLRs) and a number of cytosolic receptors detecting nucleic acid sequences indicative of viral presence within the host cell [18,19]. Once the PRRs are activated by viral molecular patterns, several transcription factors, including interferon regulatory factors (IRFs), activator protein 1 (AP-1), and NF-κB, are activated and translocated into the nucleus to induce the expression of pro-inflammatory cytokines and type I IFNs [20,21]. These secreted IFNs bind to the locally expressed IFN-α/β receptor in a paracrine and autocrine manner, and signal through the JAK-STAT pathway, which induces the expression of many IFN-stimulated genes (ISGs) [20,21]. It has been shown that fast type I IFN response protects astrocytes from flavivirus infection and virus-induced cytopathic effects [22]. IFN-inducible GTPase superfamily is also a prominent group of enzymes that operate against pathogens, and can bind and hydrolyze GTP [23]. These enzymes are involved in pathogen recognition, macrophage motility, autophagy, and phagosome maturation [24]. The well-known members of IFN-inducible GTPases that have been shown to participate in host defense include guanylate-binding proteins (GBPs) and immunity-related GTPase family M protein [23,25].

Lindqvist et al. [22] showed that the type I IFN response in astrocytes efficiently restricts the spread of both tick- and mosquito-borne neurotropic flaviviruses, including WNV, and prevents virus-induced killing of the cells. Experiments using IFN alpha receptor deficient (IFNAR−/−) astrocytes indicated that the IFN response was required for the restriction of mosquito-borne flavivirus spread in astrocytes. Lindqvist et al. [22] also showed that astrocytes from IFNAR−/− mice were more susceptible to flavivirus infection as compared to wildtype astrocytes, and this could be due to lowered basal expression of antiviral ISGs, such as viperin. Although, it has been suggested that astrocytes protect neighboring cells via IFN induction, it remains unknown if this response is generic or specific to different regions of the CNS. Thus, in this study, we used a mouse ex vivo spinal cord slice culture (SCSC) and cerebellar slice culture (CSC) models to determine similarities and differences in the innate immune responses during WNV infection of the two distinct CNS regions.

## 2. Methods

### 2.1. Cerebellar and Spinal Cord Slice Cultures

For CSC and SCSC, 7–9-day-old Brainbow/parvalbumin (PV) or GFP transgenic mouse pups were used [26]. Homozygous GFP mice were obtained from Jackson Labs, Bar Harbor, Maine. We have a colony of GFP transgenic mice in our animal facility [26]. The transgene expression is under the control of Purkinje cell specific pcp2/L7 promoter. Therefore, GFP is expressed only in Purkinje cells, where it fills dendrites, soma, axons, and nuclei. GFP fluorescence is detected in Purkinje cells as early as E17, and increases during development. Homozygous GFP mice are not very different from the wildtype animals with respect to growth, life span, and fertility. To obtain Brainbow-PV pups, we bred homozygous PV-Cre knockin mice (Jackson labs) with homozygous Thy1-Brainbow (Jackson Labs) to get distinguishable color variations in PV specific Cre recombined cells, especially in the cerebellum. PV-driven Cre expression did not affect the normal expression of PV.

All animal protocols were approved by the Institutional Animal Care and Use Committee at the University of Mississippi Medical Center. This research complies with the ‘3R’. Whole cerebellum and spinal cord was dissected out. Meninges were carefully removed using a dissection microscope. Tissue was rinsed and placed in Petri dishes containing 5% glucose and cold Gey’s balanced salt solution (Sigma-Aldrich, St Louis, MO, USA). Cerebellar and spinal cord tissues were cut into 300 µm slice sections using a McIlwain tissue chopper. Slices were suspended in cold Gey’s solution containing 5% glucose, then grown on Millicell membrane inserts (Fisher, Houston, TX, USA) using 6-well culture plate containing 1 mL plating media (*v*/*v*: 5% 10× Basal Medium with Earle’s Salt, 2.5% 10× HBSS, 25% horse serum (Invitrogen, Waltham, MA, USA), 1% 100× Pen–Strep–Glutamine, 4.5% of 10% d-glucose, 0.5% of 7.5% sodium bicarbonate, and 61.5% sterile water, Sigma, St. Louis, MO, USA). Tissue cultured plates were incubated overnight at 35.5 °C with 5% CO_2_ and 100% humidity. Old growth media was replaced by fresh media the following day followed by feeding twice a week. After confirming that cultures were healthy and viable, they were transported to a biosafety level 3 facility at the University of Southern Mississippi for WNV infection.

### 2.2. Infection with WNV

Five to seven days in vitro (DIV) old cerebellar slices were infected with 1 × 10^3^, 1 × 10^5^, or 1 × 10^7^ PFU, and the spinal cord slices with 1 × 10^3^ PFU of WNV. The infection protocol of Quick et al. [17] was followed. After 12 h exposure to WNV and an additional 72-h incubation in the normal growth media, the pooled slice cultures were processed for total RNA extraction. Total RNA collected from 3 samples (pooled)/group were analyzed for the gene expression patterns using mouse Affymetrix arrays at the Institute’s genomics and molecular core facility. Some slice cultures were fixed for immunofluorescence.

### 2.3. Immunofluorescence

Slice cultures were fixed in 4% PFA, exposed to GFP (Roche Applied Sciences, Madison, WI, USA) or GFAP (Sigma-Aldrich, St. Louis, MO, USA) antibodies, and then incubated with fluorescent secondary antibodies Alexa 488 or Alexa 546 (Invitrogen, Waltham, MA, USA). Some sections were stained with DAPI (Invitrogen) to localize nuclei. Immunostained Purkinje cells in CSC and astrocytes in SCSC were visualized under an Olympus BX60 epifluorescence microscope. Digitized images were quantified with ImageJ as previously described [26]. For statistical analysis, the Student’s unpaired *t*-test was used and the *p* < 0.05 was considered statistically significant.

### 2.4. RNA Extraction and Gene Array Analysis

Total RNA was extracted from the pooled samples containing around 12 cerebellar or 18–24 spinal cord slices/sample with RNA-extraction kit (A260/A280 ratio of 1.95 or above) or with RNA prep reagent (Trizol, ThermoFisher, Houston, TX, USA). Each RNA sample was amplified and labeled according to the Affymetrix protocol and hybridized on the Mouse 430 2.0ST GeneChip (UMMC’s Molecular and Genomics Facility). Array data were normalized, and signals were transformed to a log scale. The microarray data analysis was performed using Affymetrix^®^ Expression Console™ (Affymetrix-Thermo Fisher, Houston, TX, USA) and GeneSifter™ Software (VizX Labs, LLC, Seattle, WA, USA; http://www.genesifter.net). GeneSifter offers a robust statistical framework with 15 advanced options, including 2-way ANOVA, PCA, PAM, hierarchical clustering, etc. For statistical analysis, the Student’s *t*-test with Bonferroni’s correction was used, and the *p* < 0.05 was considered statistically significant. Genes that showed significant up- or downregulation (*p* < 0.05) with fold-changes >+1 or <−1 were identified and selected using annotation information from Gene Ontology and KEGG Pathway analysis.

## 3. Results

**Figure 1:**
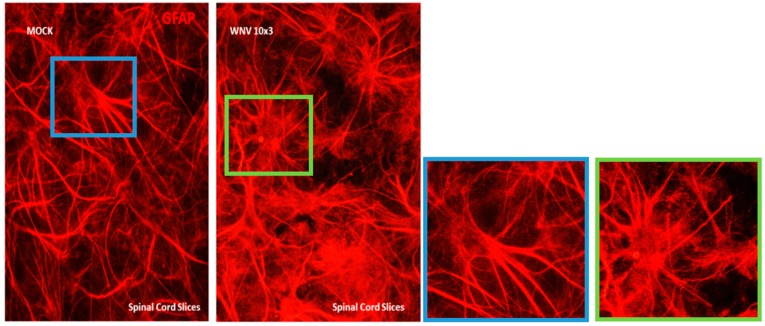
GFAP-immunostained spinal cord slice cultures (SCSCs), mock- or West Nile virus (WNV)-treated (10^3^ PFU). Compared to mock, cultures exposed to WNV showed increased GFAP immunoreactivity and marked changes in astroglial morphology (see zoomed in images in blue and green squares for mock and WNV-treated SCSC, respectively).

**Figure 2:**
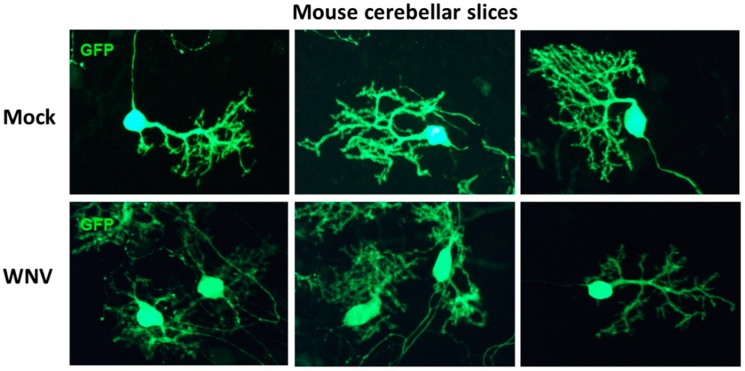
GFP immunofluorescence in mock- or WNV-treated (10^7^ PFU) cerebellar slice cultures. Since GFP localization, especially in the dendritic spines of Purkinje cells, is heterogeneous [26], the slices were immunostained with GFP antibody to enhance visualization. Purkinje cells (PCs) in WNV-exposed cultures show shrunken or pruned dendritic arbors and reduced GFP immunostaining as compared to mock cultures. The total GFP immunoreactive area of these images was quantified with ImageJ. The total area of WNV-treated PCs was significantly less (3736 ± 525.2, *n* = 5) than mock-treated PCs (9330 ± 1226, *n* = 3). The data are presented as mean ± SEM and *p* < 0.003.

We used ex vivo CSC and SCSC models to determine the innate immune response specific to the CNS during early stages of WNV infection. To infect cultures with WNV, the method of Quick and co-workers [17] was followed. Since cerebellum has a higher density of cells than spinal cord, we used 10^5^ PFU WNV/4 cerebellar slices/insert, and 10^3^ PFU WNV/6–8 spinal cord slices/insert. The inoculum was washed off at 12 h for all samples. We observed that astrocytes in mock-infected SCSCs were distributed throughout the sliced tissue and had the characteristic cellular shape (Figure 1), whereas WNV-infected samples contained reactive astrocytes, with notably enlarged cell bodies and more processes. Morphologic changes in CSCs occurred at higher doses (10^7^ PFU) of WNV, as shown in Figure 2, where Purkinje cells in WNV-exposed slices show shrunken or pruned dendritic arbors and reduced GFP immunostaining as compared to mock. The total GFP immunoreactive area of these images was quantified with ImageJ. The total area of WNV-treated Purkinje cells was significantly lesser (3736 ± 525.2, *n* = 5) than mock-treated Purkinje cells (9330 ± 1226, *n* = 3). The data is presented as mean ± SEM and *p* < 0.003. For statistical analysis, the Student’s unpaired *t*-test was used, and *p* < 0.05 was considered statistically significant.

At 3 days post-infection (dpi), Quick et al. [17] showed that WNV antigen was present in 27% of neurons and less than 11% astrocytes, and about 6% of the microglia contained WNV at 3 dpi. They further observed that by 7 dpi, the majority of neurons and a substantial percentage of astrocytes in SCSCs were infected with WNV, resulting in apoptotic cell death and astrogliosis.

**Figure 3:**
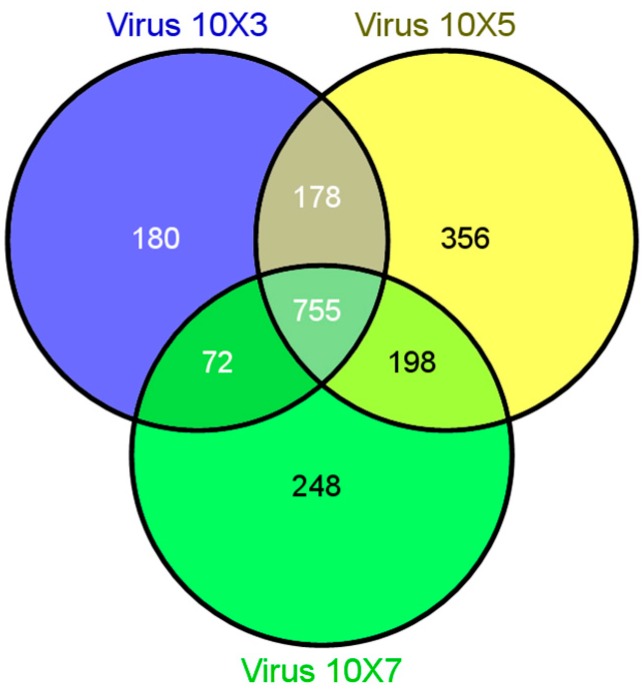
Venn diagram showing different affected genes, which are either common or unique among different treatment doses of WNV (10^3^, 10^5^ and 10^7^ PFU) in mouse cerebellar slice cultures (CSCs).

Gene expression profiling studies with mock- and WNV-treated SCSC or CSC were carried out to identify novel gene signatures related to CNS intrinsic immune response genes that modulate WNV infection. Comparison of the differently expressed genes from WNV-treated CSC revealed both common and unique transcripts across 10^3^, 10^5^, and 10^7^ PFU treatment doses of WNV (Figure 3). 

**Figure 4:**
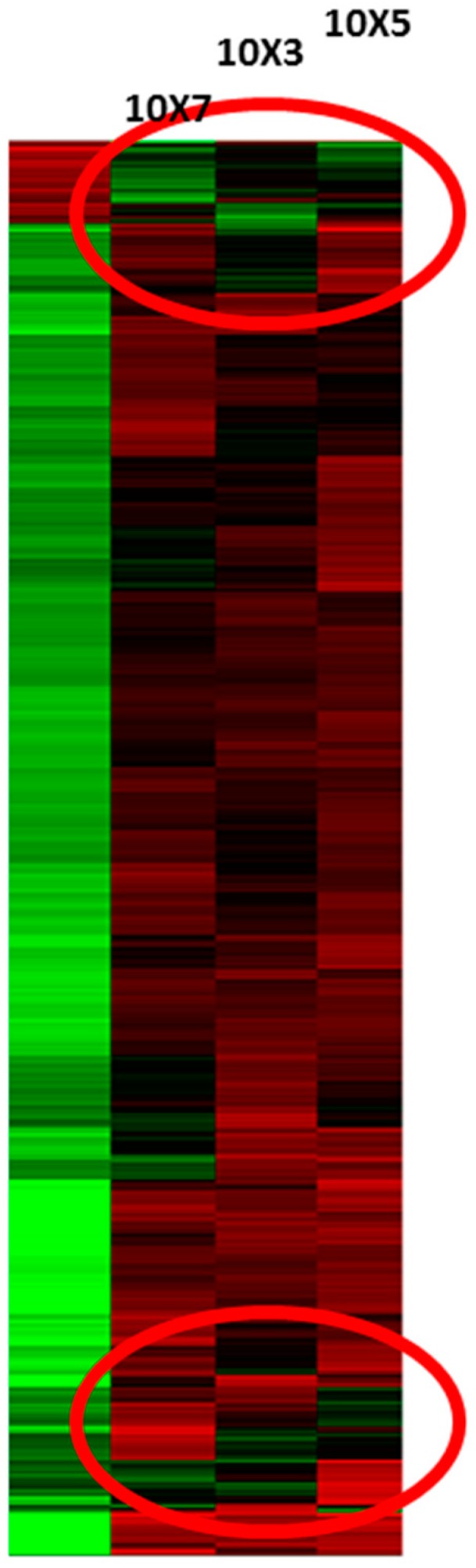
Hierarchical gene clusters (red circles) of expressed genes after treatment with different doses of WNV in cerebellar slice cultures. The left-most column represents mock treatment.

Figure 4 shows hierarchical clustering of genes with altered expression level after treatment with different doses of WNV in CSC. The rapid triggering of an IFN-α/β response results in the early control of flavivirus infection in mammalian cells.

In the early phase of infection, viral nucleic acid sensing induces nuclear localization of IRFs, which stimulate gene transcription and production of IFN-α/β by infected cells. In the later phase, these IFNs bind to the common IFN-α/β receptor in a paracrine and autocrine manner and signal through the JAK-STAT pathway, resulting in the induced expression of hundreds of ISGs. Viperin is one of the ISGs with antiviral activity against WNV [20,21]. Some of the highly upregulated ISGs in post-WNV-infected CSC are listed in Table 1. The upregulation was dose dependent.

Further, for statistical analysis with sample size of three (*n* = 3) per group, total RNA was collected by pooling 9 CSCs or 18–24 SCSCs (WNV treated or mock) per sample, and were subjected to gene expression profiling using mouse Affymetrix arrays. The Student’s *t*-test with Bonferroni’s correction (*p* < 0.05) revealed that, in SCSCs, 6884 genes were upregulated and 2756 were downregulated. By contrast, only 2756 genes were upregulated and 3856 downregulated in the CSCs (Table 2, Table 3 and Table 4).

The expression patterns of a number of genes were significantly altered between the mock- and WNV-treated groups both in the CSCs and SCSCs (Figure 3 and Figure 4). However, there were distinct differences, such that CSCs showed robust induction of interferons (IFNs), IFN-stimulated genes (ISGs), and interferon regulatory factors (IRFs), which was not seen in SCSCs. Gene sets related to many other cellular processes were also affected. Additionally, numerous genes specific to non-protein-coding mRNAs were found (GEO accession number GSE123793). However, in this manuscript, we are only reporting alterations in the interferon-related genes and chemokines and/or cytokines.

**Figure 5:**
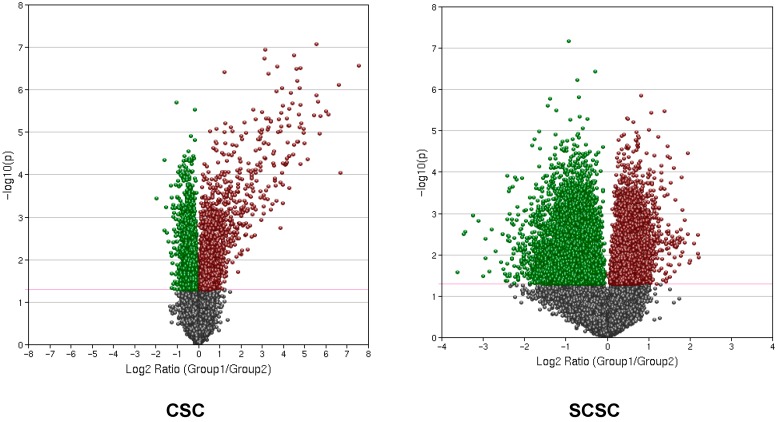
A volcano plot showing relationship between fold-change of gene expression (CSC; SCSC) and statistical significance. The *x*-axis displays magnitude of fold-change on a log scale and *y*-axis displays statistical significance −log10 of *p* value. The magenta line shows *p* = 0.05 cut-off, with points above the line falling in *p* < 0.05 region and below the line in *p* > 0.05 region. The red data points indicate upregulation and the green indicate down regulation. The points having a fold-change less than 2 (log2 = 1) are shown in gray.

We used volcano plots to assess the magnitude of fold-change of gene expression to statistical significance [28,29], which allowed us to find genes of interest and then compares and contrasts the data of CSC with SCSC (Figure 5). The volcano plot arranges genes along two dimensions. The horizontal dimension is the fold-change between the two groups on a log scale, and the vertical dimension represents the *p*-value for a *t*-test of differences between samples represented by a negative log scale (−log10 of *p* value), thus smaller *p*-values are placed higher up. The magenta line shows *p* = 0.05 cut-off with points above the line falling in the *p* < 0.05 region and points below the line in the *p* > 0.05 region (Figure 5). The red points indicate upregulated genes and green downregulated genes. Gray indicates points with a fold-change less than 2 (log2 = 1).

Clarke et al. [27] showed that the intracerebral infection of the mouse brain with WNV led to the differential regulation of many genes within the CNS. They used Ingenuity Pathway Analysis and reported 23 IFN signaling genes with the highest levels of upregulation (50-fold), which included genes expressing GTPases, antiviral proteins, immune cell attractants, and proteins involved in the regulation of IFN signaling. We compared our data with Clarke et al. [27] and presented it in Table 3 and Table 4. CSCs showed robust induction of interferons (IFNs), IFN-stimulated genes (ISGs), and regulatory factors (Table 3). Some of the antiviral genes related to IFN were upregulated more than 25-fold in CSCs as compared to mock. By contrast, though SCSCs had twice the number of dysregulated genes as compared to CSCs, they exhibited much subdued induction of ISGs (Table 4). Clarke and co-workers [27] also used brain slice culture to examine WNV-induced pathogenesis in the absence of a peripheral immune response and suggested that WNV-induced neuronal injury in the brain is mediated by death receptor-induced apoptosis. IFN-induced apoptosis gene lipocalin2 (Lcn2) was upregulated 56-fold. In our CSC and SCSC, Lcn2 was upregulated <4-fold and <2-fold, respectively, indicating that apoptosis may not be the primary pathway influencing WNV pathogenesis in our models.

**Figure 6:**
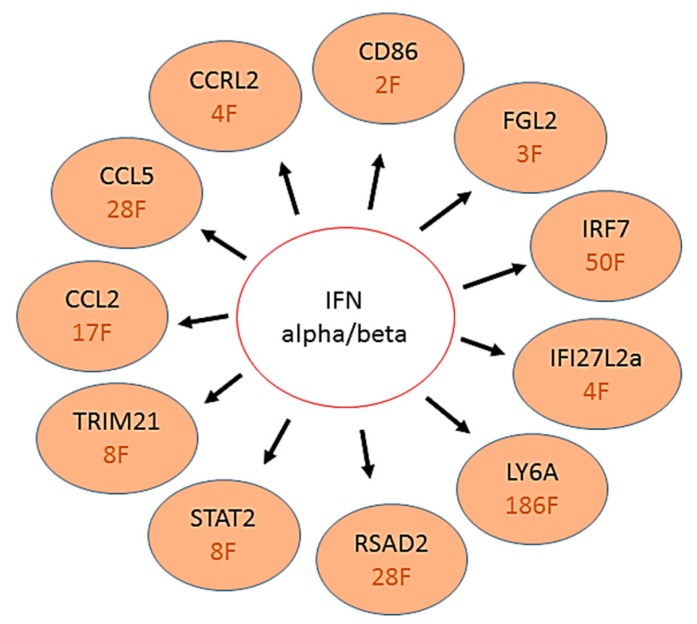
Schematic showing IFN-α/β activation and induction of related ISGs or IFN regulators in CSCs. The red text indicates fold (F) upregulation.

Lindqvist et al. [22] reported that astrocytes respond very quickly after viral infection by upregulation of type I IFNs. This upregulation restricts virus replication and spread in primary cultures, and contributes to cell survival. We looked at some of the upstream activator genes proposed by Lindqvist et al. [22] that were significantly upregulated in their astrocytic primary cultures (Figure 6). Interestingly, many similar IFN-α/β upstream activator genes were upregulated by several fold in CSC (Figure 6). By contrast, except FGL2 (1.5-fold) and CCRL2 (1.1-fold), none of the other genes listed in Figure 6 were significantly different as compared to mock in SCSC, which suggests that the cerebellar microenvironment may have stronger innate antiviral defenses than spinal cord. Our recent data (unpublished) is supportive of this argument. Mice chronically exposed to a single sublethal dose of WNV showed spinal cord susceptibility and a much higher number of dysregulated genes than cerebellum. We believe that most of the ISGs or regulatory factors upregulated in WNV-infected CSC are specifically associated with the astrocytic population of those slice cultures.

## 4. Discussion

We used two ex vivo slice culture models to compare and contrast the early intrinsic CNS immune response of resident cells in different CNS regions during WNV infection. The rationale for using ex vivo models was that slice cultures are able to maintain the cytoarchitecture of the CNS, which allows a thorough understanding of the functions of multiple interconnected cells in a culture system that closely resembles the in vivo environment [17]. Additionally, whereas experimental mouse models of WNV infection were able to show the importance of innate and adaptive immune responses in controlling the extent and severity of CNS disease, they could not clearly differentiate immune responses intrinsic to the CNS from those that are dependent on infiltrating inflammatory cells [17,30,31,32].

To understand the early intrinsic anti-inflammatory response in the CNS, we looked at the effects of WNV infection as early as 3 dpi. Quick and co-workers [17] also reported that at 3 dpi, only 27% of neurons, less than 11% astrocytes, and about 6% microglia were infected with WNV. In another study, Clarke et al. [27] investigated WNV-induced injury to brain slice cultures by determining the levels of lactate dehydrogenase (LDH) in the media of WNV-infected cultures compared to mock-infected controls. No LDH leak was detected at 3 dpi, suggesting negligible injury to the brain slice cultures at 3 dpi [27]. Further, at 3, 5, 7, and 9 dpi, caspase 3 activity assays were used to determine the amount of WNV-induced apoptosis in brain slice cultures. At 3 dpi, the fold increase in caspase 3 activity infected vs. mock was not significant. We also did not see any upregulation of caspase 3 gene expression either in CSC or SCSC. At 3 dpi, mild astrogliosis was seen in our SCSCs (Figure 1) and Purkinje cell damage was visible only in 10^7^ PFU WNV-treated CSCs (Figure 2).

Adult mice infected with 100 PFU WNV by intracerebral inoculation [27] showed 50-fold upregulation of about 23 genes involved in IFN signaling, including genes expressing GTPases, antiviral proteins, immune cell attractants, and proteins involved in the regulation of IFN signaling. Several antiviral ISGs, including viperin, have been identified as inhibitors of WNV, and are also downregulated in IFNAR−/− astrocytes, and upregulated after treatment with IFNαB/D and supernatant [22], and could, thus, contribute to antiviral response against WNV in astrocytes [33,34,35]. In addition, Lindqvist et al. [22] were able to predict upstream regulators responsible for the differential expression patterns in IFNAR−/−-, supernatant-, and IFNαB/D-treated astrocytes, and identified type I IFN, as well as IFN signaling molecules, to have the highest activation scores. IFNγ-STAT1-IRF-1 signaling cascade was predicted as an upstream regulator both in supernatant and IFNαB/D-treated astrocytes [22]. Similar observations were made in our study, where IFN signaling and antiviral mediators were among the most upregulated genes in WNV-infected CSCs (Table 1 and Table 3; Figure 6).

Mammalian cells have been shown to detect WNV and induce type 1 IFNs during the earliest stages of WNV infection after host cell recognition of viral RNA, and mice with genetic defects in the receptor for IFN-α/β, or constituents of its signaling cascade, show markedly enhanced viral accumulation in tissues, leading to rapid lethality [36]. WNV has evolved to counter IFN function of restricting viral infection and to limit its efficacy. It also attenuates IFN function at multiple steps of the induction and signaling cascade [37,38,39,40,41,42]. Indeed, WNV is resistant to the antiviral effects of IFN in cell culture once infection is established, which may explain the relatively narrow therapeutic window for IFN administration that has been observed in animal models or humans infected with WNV [43].

Viperin plays an important player in mediating the IFN-dependent antiviral response and inhibiting a number of viruses at various stages of their life cycles [44,45,46]. A few studies have sought to define the function of viperin in vivo. For WNV, higher viral burdens were observed in the spleen, kidney, and brain in the absence of viperin, which suggests greater susceptibility of viperin−/− mice to infection [33]. Astrocytes are important producers of IFNs during neurotropic viral infections [47,48]. Lindqvist et al. [22] have shown that IFN signaling controls flavivirus infection and that viperin is highly upregulated in astrocytes. They further demonstrated that WNV infection was significantly reduced after 48 hpi in viperin−/− cortical neurons treated with IFN, indicating that other ISGs also contribute to the IFN-mediated antiviral responses against WNV [46]. Furthermore, the antiviral effects of viperin are restricted to the specific brain regions [46]. Our data also show 28-fold and dose-dependent upregulation of viperin in CSCs (Table 1 and Table 3; Figure 6), however, no change in viperin gene expression was seen in SCSC. We speculate that viperin mediates CNS region- and cell-type-specific inhibition of WNV. Our results suggest that early anti-inflammatory response to WNV infection in CSCs may possibly be due to a large population of specialized Bergmann astroglial cells in the cerebellum. We further speculate that lack of such specialized astrocytes in SCSC may make spinal cord more susceptible to WNV damage. Indeed, this finding may help to explain the clinical susceptibility of grey matter of human spinal cord to WNV infection. The most common neuromuscular manifestation of WNV infection is an acute flaccid paralysis attributed to a poliomyelitis syndrome with asymmetric paralysis variably involving one (monoparesis) to four limbs (quadriparesis), with or without brainstem involvement and respiratory failure [9,49]. This syndrome of acute flaccid paralysis may occur without overt fever or meningoencephalitis. Involvement of anterior horn cells in the spinal cord is the major site of pathology responsible for the neuromuscular deficits.

In sum, our results suggest that early anti-inflammatory response to WNV infection in CSCs may be due to large population of distinct astrocytic cell types, and lack of those specialized astrocytes in SCSC may make spinal cord cells more susceptible to WNV damage.

## 5. Conclusions

The use of ex vivo slice cultures of cerebellum and spinal cord tissue has enabled us to determine similarities and differences between the innate immune responses of the resident CNS cells from different regions, especially the response of astrocytes to WNV infection. Since no effective treatment strategy is currently available for managing WNV neuroinvasive disease, the understanding of early neuroprotective or pathologic events in WNV-infected ex vivo culture models could help develop potential therapeutics.

## Figures and Tables

**Table 1:** Dose-dependent upregulation of the selected interferon (IFN)-induced and associated genes in mouse CSCs infected with WNV. CSCs were treated with 10^3^, 10^5^, or 10^7^ PFU of WNV.

Gene Name	Gene Designation	10^3^ PFU	Fold Upregulation 10^5^ PFU	10^7^ PFU
Interferon-induced protein 44	Ifi44	127	162	156
Lymphocyte antigen 6 complex locus A	Ly6a	80	114	136
Interferon-induced protein with tetratricopeptide repeats	Ifit1	29	37	40
Viperin	Rsad2	24	29	33

**Table 2:** WNV-induced gene regulation in mouse CSCs and SCSCs. Number of genes up- or downregulated. Total number of dysregulated genes.

Gene Category	Cerebellum (Total)	Spinal Cord (Total)
Upregulated	2756	6884
Downregulated	3856	5853

Microarray analysis was performed on RNA extracted from the mouse cerebellar and spinal cord slices infected with WNV. The data (*n* = 3 per group) were analyzed using Student’s *t*-test with Bonferroni’s correction. *p* < 0.05 was considered statistically significant.

**Table 3:** Genes associated with IFN signaling are highly upregulated in the mouse CSCs infected with WNV. The data (*n* = 3) were analyzed using Student’s *t*-test with Bonferroni’s correction (*p* < 0.05 statistically significant).

Function	Gene Designation	Name	Fold Upregulation	*p* Value
Chemoattractant for immune cells	Cxcl10	C–X–C motif chemokine 10	22.86	0.0045
	Ccl5	Chemokine (C–C motif) ligand 5	27.82	5.47 × 10^−5^
IFN-inducible GTPase	Irgm1	Immunity-related GTPase family M member1	12.82	1.08 × 10^−6^
	Irgm2	Immunity-related GTPase family M member2	21.35	2.03 × 10^−6^
	Gbp2	Guanylate-binding protein-2	26.40	3.73 × 10^−6^
	Gbp6	Guanylate-binding protein-6	20.26	1.19 × 10^−6^
Regulation of IFN signaling	Usp18	Ubiquitin-specific peptidase 18	43.58	3.94 × 10^−6^
	Nlrc5	NLR family, CARD domain containing 5		
	Ifna2	Alpha interferon 2	1.92	0.0235
Acute-phase response	Saa3	Serum amyloid A 3	3.88	0.0071
IFN-induced antiviral activity	Rsad2	Viperin (radical *S*-adenosyl methionine domain-containing 2)	28.11	3.09 × 10^−7^
	Ifit1	IFN-induced protein with tetratricopeptide repeats1	47.51	8.25 × 10^−8^
	Ifit2	IFN-induced protein with tetratricopeptide repeats2	9.08	4.07 × 10^−6^
	Osal2	2′–5′ oligoadenylate synthase-like 2	52.36	1.09 × 10^−5^
IFN-induced, unknown function	Slfn4	Schafen 4	2.67	0.00047
	Ifi44	Interferon-induced protein 44	96.71	7.67 × 10^−7^
IFN-induced apoptosis	Lcn2	Lipocalin2 [27]	3.65	0.00026

**Table 4:** Genes associated with IFN signaling are altered in the mouse spinal cord slice cultures infected with WNV.

Function	Gene Designation	Name	Fold-Change	*p* Value
Chemoattractant for immune cells	Cxcl10	C–X–C motif chemokine 10	1.52↑	0.0344
IFN-inducible GTPase	Gbp6	Guanylate-binding protein-6	1.91↓	0.0112
IFN-induced apoptosis	Lcn2	Lipocalin2	1.66↑	0.0347
